# Effects of Measurement Distance on Linear Taylor Patterns with Reduced Inner Sidelobes

**DOI:** 10.3390/s25185803

**Published:** 2025-09-17

**Authors:** Aldara Seoane-Campos, María Elena López-Martín, Juan Antonio Rodriguez-Gonzalez, Francisco Jose Ares-Pena

**Affiliations:** 1 Radiating Systems Group, Department of Applied Physics, University of Santiago de Compostela, 15782 Santiago de Compostela, Spain; aldara.seoane@rai.usc.es (A.S.-C.); ja.rodriguez@usc.es (J.A.R.-G.); 2 Department of Morphological Sciences, University of Santiago de Compostela, 15782 Santiago de Compostela, Spain; melena.lopez.martin@usc.es

**Keywords:** antenna, line sources, Linear Taylor distributions, measurement distance

## Abstract

The influence of distance on Taylor diagrams with one, two, and three depressed inner lobes was analyzed in the context of linear distributions. High sidelobes are tolerated in these patterns, except in the case of the inner lobes, which are positioned at a significantly lower level to minimize interference and optimize efficiency. The classical method described by Elliott was used to compute the necessary roots in the Taylor distribution. The study was conducted considering $\bar{n}$ equal to 6 and a sidelobe level (SLL) of −20 dB for all lobes except the first inner positioned at −40 dB.

## 1. Introduction

The electromagnetic radiation field is conventionally divided into two primary regions: the far-field and the radiative near-field. The separation between these regions is defined by the Rayleigh distance, also referred to as the Fraunhofer distance [1]. The precise distance at which this phenomenon occurs is contingent on the product of the square of the antenna aperture and the carrier frequency, as previously outlined by [1]. In the region beyond the Rayleigh distance, the electromagnetic waves are approximated as plane waves in the far-field region. Conversely, within this distance, near-field effects predominate, necessitating a more precise representation of the electromagnetic field by using spherical waves. A recent revision has been presented in [2] regarding the electromagnetic field zones and the related analytical derivations. The concept of far-field distance has been generalized for various applications in [3,4,5].

The term line source denotes any radiating structure characterized by an elongated, narrow, straight geometry, wherein the pattern characteristics arise from variations in current or field intensity along the longitudinal axis. This investigation focuses on continuous line sources, defined by currents or fields that are continuous functions of the longitudinal coordinate [6]. It is important to emphasize that the analysis of arrays comprising numerous discrete elements often commences by with the consideration of a continuous distribution enveloping the discrete function [7].

Methodologies for synthesizing line source aperture distributions that yield antenna radiation patterns featuring a narrow main lobe accompanied by symmetric and low-level sidelobes originate from prior studies on nonuniformly excited linear arrays with equispaced discrete elements. Dolph [8] exploited a distinctive property of Chebyshev polynomials: their formation as sequences of uniformly oscillating functions followed by hyperbolic growth. By applying this property to the design of linear arrays of discrete elements, Dolph identified an optimal distribution that minimized beamwidth for a specified sidelobe level. Building on Dolph’s findings, Taylor [6] demonstrated that the continuous limit of the Dolph–Chebyshev aperture distribution is physically unattainable due to the excitation magnitudes at the aperture edges tending toward infinity. Subsequently, Taylor derived a distribution that produces a radiation pattern with a narrow main beam and symmetric sidelobes closely approximating the Dolph pattern without requiring infinite excitations at the edges.

The Taylor line source distribution is based on adjusting the positions of the inner-pattern zeros (nulls) of a uniform distribution, thereby generating a distribution that produces the desired radiation pattern. A predetermined number of sidelobes on each side of the main beam are maintained at an approximately uniform level, while sidelobes located farther from the main beam gradually decrease in amplitude.

Linear Taylor distributions play a pivotal role in the synthesis of equispaced linear arrays with a large number of discrete elements, as has been demonstrated by sampling the corresponding continuous line sources [7]. Hansen conducted studies on sum patterns for linear Taylor distributions, particularly focusing on $\bar{n}$ monotonic distributions, to investigate the dependence on distance (normalized to the Rayleigh distance) of the field in patterns with low sidelobe levels [9]. A concomitant study revealed that using optimal $\bar{n}$ values does not substantially enhance the efficiency, a finding that corroborates Hansen’s observations [10]. Taylor distributions can also be applied to the design of equally spaced linear arrays. By calculating the excitation at as many points as there are elements, the resulting radiation pattern approaches the theoretical ideal as the number of elements increases. However, when the number of elements is small, the resulting pattern suffers from significant degradation.

Hansen’s research was also extended to the examination of Bayliss difference patterns [11] which are characterized by low sidelobe levels and monotonic $\bar{n}$ values. Additional studies have explored shaped patterns generated by line sources, as presented in [12].

Concurrently, contemporary radar architectures impose mounting constraints on pattern characteristics. Specifically, the demand for reduced sidelobe levels requires enhanced precision in directing the main beam, thereby improving detection accuracy and overall system performance. This need has spurred the development of various methods tailored to environments with uneven signal distributions. In this regard, some approaches have been applied to circular distributions, as detailed in [13,14]. The efficacy of these methods in enhancing pattern synthesis has been demonstrated for particular scenarios.

In the landscape of sixth-generation (6G) mobile networks, extremely large-scale antenna arrays (ELAAs) have emerged as a foundational technology. These networks form the basis of advanced systems such as ultra-massive multiple-input multiple-output (UM-MIMO), cell-free massive MIMO [15], reconfigurable intelligent surfaces (RIS), and terahertz communications. Given the sheer scale of antennas in ELAAs, the traditional planar-wave model used in 5G massive MIMO is insufficient. Instead, accurate modeling requires near-field spherical wave propagation. This shift highlights the critical role of near-field MIMO communications in the development of 6G wireless networks [16,17,18,19].

In the study cited in [13], ϕ-symmetric Taylor diagrams with suppressed lobes were constructed for circular distributions. In addition, the null-filling effects over measurement distances on circular Taylor patterns have been studied in [14]. However, no comparable analysis has been conducted for linear distributions, which can be used for the synthesis of planar arrays with a large number of elements and that do not exhibit ϕ-symmetric radiation patterns. The present study focuses on linear distributions, investigating variations in efficiency when one suppressed lobe and two suppressed lobes are maintained. Efficiency was defined here as the ratio between the maximum directivity of an antenna aperture and of a uniformly excited distribution of equivalent size. The objective of this study was to explore these distributions and their potential for optimizing antenna efficiency.

## 2. Materials and Methods

**Figure 1:**
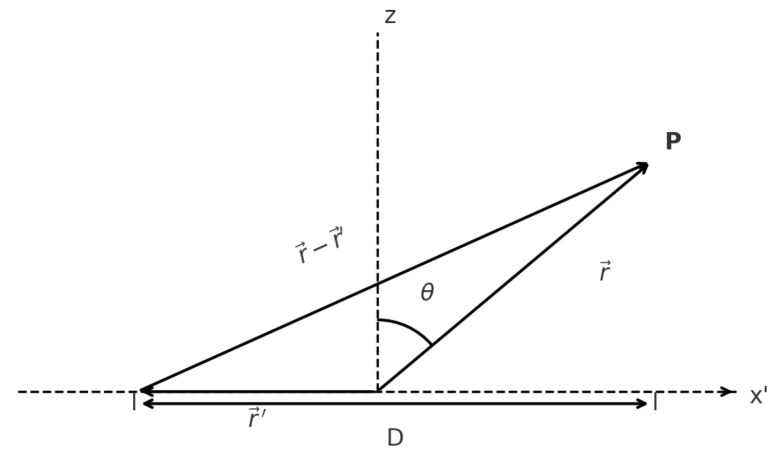
Geometry of the problem.

In this work, we consider a linear aperture of length Dλ, as shown in Figure 1, operating in the Fresnel region, where near-field effects are significant and must be taken into account in the analysis of the radiated field.

The general expression for a linear aperture in the Fresnel region, as presented by Hansen [20] and Walter [21], is given by

(1) $$F\left(r,\theta \right)=A_{1}\int_{D}I\left(r^{\prime }\right)e^{-jk\left|r-r^{\prime }\right|}\;dr^{\prime }$$

As shown in Figure 1, the origin of coordinates is placed at the center of the linear aperture. The position vector of the observation (field) point, measured from the center of the aperture, is denoted by r, while the position vector of a current element along the aperture is denoted by $r^{\prime }$. The excitation of the current element at position $r^{\prime }$ is given by $I(r^{\prime })$. The wavenumber is defined as $k=\frac{2\pi }{\lambda }$, where λ is the wavelength. The angle θ is defined as the angle between the position vector r and the *z*-axis (angle from broadside). The constant $A_{1}$ is a multiplicative factor that does not affect the field distribution.

By performing a Taylor series expansion of $\left|r-r^{\prime }\right|$ in powers of $\frac{x^{\prime }}{r}$, where $x^{\prime }$ is the algebraic distance along the aperture measured from its center, and neglecting terms of order ${\left(\frac{x^{\prime }}{r}\right)}^{n}$ with n≥3, we obtain

(2) $$\left|r-r^{\prime }\right|\approx r\left[1-\left(\frac{x^{\prime }}{r}\right)\sin\theta +\frac{1}{2}{\left(\frac{x^{\prime }}{r}\right)}^{2}-\frac{1}{2}{\left(\frac{x^{\prime }}{r}\right)}^{2}\sin^{2}\theta \right]=$$

(3) $$r-x^{\prime }\sin\theta +\frac{1}{2r}x^{\prime 2}-\frac{1}{2r}x^{\prime 2}\sin^{2}\theta$$

Introducing the following change in variables:

$$k=\frac{2\pi }{\lambda },\;\sin\theta =w,\;u=\frac{wD}{\lambda },\;p=\frac{2x^{\prime }}{D},\;\gamma =\frac{r}{\left(\frac{2D^{2}}{\lambda }\right)}$$

(4) $$k\left|r-r^{\prime }\right|\approx \frac{2\pi }{\lambda }r-\pi up+\frac{\pi p^{2}}{8\gamma }-\frac{\pi P^{2}}{8\gamma }w^{2}$$

It is evident that, since the radius of convergence—denoted by *r*—is finite, an approximation error arises. This error depends on the distance *r* and, consequently, on the parameter γ (normalized measurement distance). However, given that the magnitude of *w* is small within the region of the first sidelobe, the term $w^{2}$ may be neglected. This simplification is justified by the fact that the primary concern is the error affecting the sidelobe level, which is predominantly influenced by the first sidelobe.

The far-field spatial pattern corresponding to the Taylor $\bar{n}$ factor is commonly expressed as follows [10]:

(5) $$F\left(u\right)=\frac{\sin\pi u}{\pi u}\prod_{n=1}^{\bar{n}-1}\frac{1-\frac{u^{2}}{z_{n}^{2}}}{1-\frac{u^{2}}{n^{2}}}$$

but is alternatively written as a sum of 2$\bar{n}$ − 1 sinc beams:

(6) $$F\left(u\right)=\sum_{n=-\bar{n}+1}^{\bar{n}-1}F_{n}\;\sin c\;\pi \left(u+n\right)$$

The aperture distribution for a Taylor line source is given by [9,10]:

(7) $$g\left(p\right)=\frac{1}{2}\sum_{n=-\bar{n}+1}^{\bar{n}-1}F_{n}e^{j\pi np}$$

The coefficients $F_{n}$ are then defined as follows:

(8) $$F_{n}=\frac{{\left[\left(\bar{n}-1\right)!\right]}^{2}}{\left(\bar{n}+n-1\right)!\left(\bar{n}-n-1\right)!}\prod_{m=1}^{\bar{n}-1}\left(1-\frac{n^{2}}{z_{m}^{2}}\right)$$

(9) $$F_{0}=1$$

The zeros are defined as

(10) $$z_{n}=\pm n,\;n\geq \bar{n}$$

(11) $$z_{n}=\pm \sigma \sqrt{A^{2}+{\left(\bar{n}-\frac{1}{2}\right)}^{2}},\;1\leq n\leq \bar{n}$$

and the dilation factor is

(12) $$\sigma =\frac{\bar{n}}{\sqrt{A^{2}+{\left(\bar{n}-\frac{1}{2}\right)}^{2}}}$$

It is evident that the space factor contains zeros beyond $\bar{n}$ at the integers, with the first $\bar{n}-1$ zeros modified to adjust the first $\bar{n}-1$ sidelobes.

Let *A* be the measure of the sidelobe level (SLL), where the relationship is given by the following equation:

(13) cosh(πA)=b

and

(14) $$20\log_{10}b=\mathrm{SLL}$$

As a result of the proposed approach, the initial function is reformulated as follows, taking into account that $I\left(r^{\prime }\right)=I\left(x^{\prime }\right)=g\left(p\right)$.

(15) $$F\left(r,u\right)=\frac{D}{2}A_{1}\int_{-1}^{1}g\left(p\right)\;e^{-j\frac{2\pi }{\lambda }r}\cdot e^{-j\left(-\pi up+\frac{\pi p^{2}}{8\gamma }\right)}\;dp$$

(16) $$F\left(r,u\right)=A_{2}\int_{-1}^{1}g\left(p\right)\;e^{-j\left(-\pi up+\frac{\pi p^{2}}{8\gamma }\right)}\;dp$$

where $A_{2}=\frac{D}{2}A_{1}\;e^{-j\frac{2\pi }{\lambda }r}$ is also a constant that does not affect the radiation pattern, so we can exclude it from the expression.

In the far-field approximation, we consider the observation point at infinity, i.e., r→∞. Therefore, the parameter γ, which is a function of *r*, also tends to infinity: γ→∞. Then Equation (15) becomes

(17) $$F\left(r,u\right)=F\left(\gamma ,u\right)=\int_{-1}^{1}\frac{1}{2}\sum_{n=-\bar{n}+1}^{\bar{n}-1}F_{n}\;e^{j\left(\pi (n+u)p\right)}\;dp$$

Using $\beta =\frac{\pi }{8\gamma }$ in (16), which represents the edge phase error, we arrive at the final expression, which is consistent with the formulation presented by Hansen in [9]:

(18) $$F\left(r,u\right)=\frac{1}{2}\sum_{n=-\bar{n}+1}^{\bar{n}-1}F_{n}\int_{-1}^{1}e^{-j[p^{2}\beta -\pi \left(n+u\right)p]}dp$$

where $\bar{n}-1$ represents the number of pattern roots used to control the depression of sidelobes on each side of the Taylor pattern.

To obtain sum patterns with “arbitrary sidelobe topography,” the method discussed in [22] and in [7], was used to determine the zeros $Z_{n}$ in Equation (5). Based on this approach, the peak directivity of the sum pattern produced by a far field continuous line source is given by

(19) $$D\left(\theta \right)=\frac{F\left(\theta_{0}\right)F^{*}\left(\theta_{0}\right)}{\frac{1}{4\pi }\int_{0}^{2\pi }\int_{0}^{\pi }F\left(\theta \right)F^{*}\left(\theta \right)\sin\theta \;d\theta \;d\phi }$$

where the denominator represents the total power radiated by the line source over a large sphere centered at the aperture. The numerator corresponds to

(20) $$P\left(\theta \right)=F\left(\theta \right)F^{*}\left(\theta \right)$$

where $\theta_{0}$ denotes the direction of peak radiation.

Due to symmetry, Equation (6) simplifies to the following:

(21) $$D\left(\theta_{0}\right)=\frac{2P(\theta_{0})}{\int_{0}^{\pi }P\left(\theta \right)\sin\theta \;d\theta }$$

To normalize the peak directivity, the same procedure is applied to the expression corresponding to a uniform continuous line source. This yields the following normalization factor used for efficiency calculations (which corresponds to the maximum directivity of an aperture antenna relative to its standard directivity, where the standard directivity refers to the maximum directivity achievable by a linear source of length *D*, when excited with a uniform-amplitude and in-phase distribution; in our case, this is referred to the directivity of the uniform distribution) [23,24]:

(22) $$\eta =\frac{D(\theta_{0})}{D_{\mathrm{uniform}}\left(\theta_{0}\right)}$$

## 3. Results

In this section, we use Equations (8) and (18), outlined in the Methods section, to describe the near-field patterns of symmetric linear Taylor distributions. Initially, we compared peaked and monotonic distributions, analyzing the differences in their shapes and their effect on far-field measurements.

For linear Taylor distributions, there is no significant difference between the peaked and monotonic cases, as demonstrated in [10]. We therefore focused on the $\bar{n}$ monotonic distribution with a sidelobe level (SLL) of −40 dB. We then attempted to reconstruct the near-field patterns while allowing for errors of 1.0 dB, 0.5 dB, and 0.1 dB. The results of these reconstructions are presented in Table 1, and will be important in the comparative analysis in the section below.

In all figures, two representations of the function were used. The figure corresponding to γ=1000 is depicted by a dashed line, and the figure with degraded performance (γ=0.5) is depicted by a solid line.

We reproduced the values from [9] using the same error margins, which will later be compared with the suppressed lobes.

In the following examples, we use Elliott’s method [22], which is based on an iterative root design approach that enables adjustment of a specified number of sidelobes to a desired level. As a result, we no longer use Equation (11) from the conventional Taylor root calculation.

We present the results of the root calculation (which were used to generate the figures) in Table 2. These figures formed the basis of our study on radiation pattern reconstruction. The reconstruction was carried out using the same error margins as in the previous section.

### Extension to Large Rectangular Apertures

Although this article focuses on linear aperture distributions, the results obtained can also be applied to separable rectangular apertures [21,25,26]. A widely used approach in the synthesis of large planar antennas is this method, which assumes that the aperture distribution can be represented as the product of two orthogonal linear distributions—aligned along the *x*- and *y*-axes—such as those analyzed in this work. Consequently, the aperture function can be expressed as

$$g_{a}\left(x,y\right)=g_{x}\left(x\right)\cdot g_{y}\left(y\right)$$

This assumption simplifies the analysis by decoupling the two dimensions, allowing for the radiation pattern to be described independently in each principal plane.

As a result, the radiation patterns in the principal planes coincide with the field generated by those distributions, while the patterns in the intercardinal planes correspond to the product of both.

A typical limitation of this method is the non-uniform sidelobe behavior: sidelobes tend to be more pronounced along the principal planes, and significantly attenuated in other directions.

**Figure 2:**
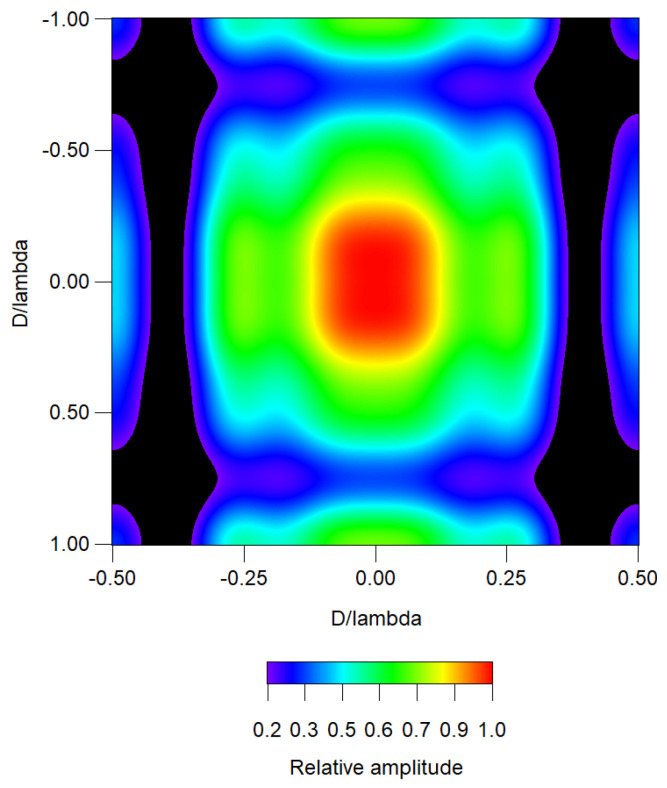
Interpolated image of a separable rectangular distribution with a 1:2 aspect ratio.

Figure 2 shows the separable planar distribution obtained from the combination of two linear distributions previously analyzed in this article: one with a suppressed lobe along the *x*-axis, and another with three suppressed lobes along the *y*-axis. This is an interpolated image representing a rectangular distribution with a 1:2 aspect ratio.

## 4. Discussion

In the four study cases, the efficiency was lowest, with a value of 0.7729, when all sidelobes were suppressed to −40 dB. The loss of efficiency is attributed to the greater restriction imposed on the entire radiation pattern, resulting in greater dispersion of energy outside the main lobe. We attempted to recover the field by allowing certain errors, (Table 3).

**Figure 3:**
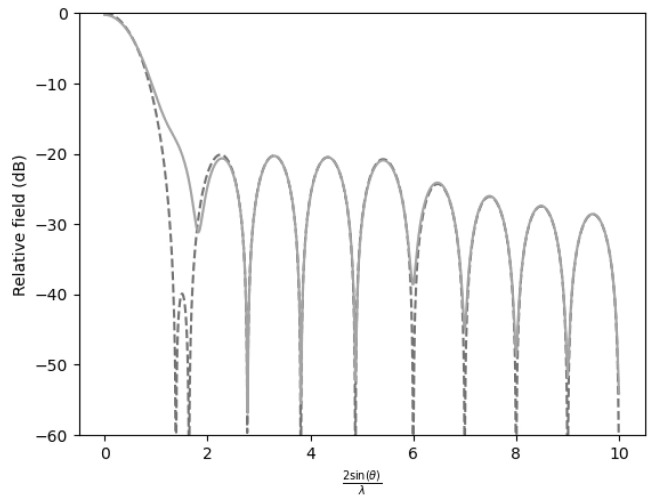
Comparison of the far-field expression (γ=1000) with the near-field expression (γ=0.5) for −20 dB SLL with $\bar{n}=6$ and the first sidelobes at −40 dB.

Conversely, when only a single sidelobe was suppressed to −40dB, (Figure 3), the efficiency reached a maximum level of 0.9084. This suggests that allowing for most sidelobes to remain at less restrictive levels enhances concentration of energy in the desired direction. In summary, the efficiency penalty is minimal when only one sidelobe is attenuated.

**Figure 4:**
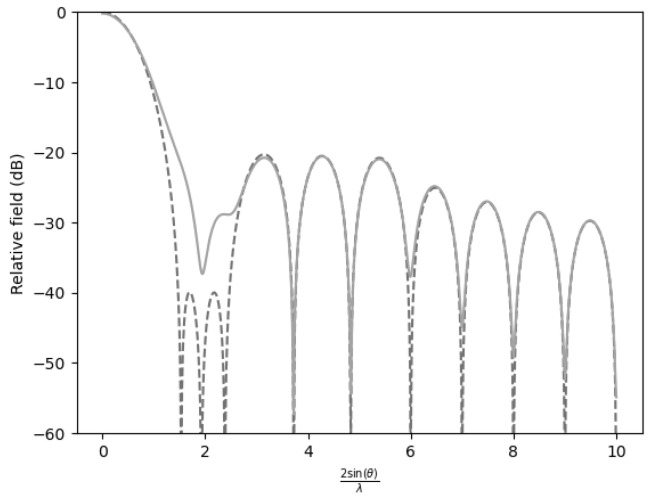
Comparison of the far-field expression (γ=1000) with the near-field expression (γ=0.5) for −20 dB SLL with $\bar{n}=6$ and the first two sidelobes at −40 dB.

Finally, when two sidelobes were suppressed to −40dB while the remaining sidelobes were maintained at −20dB, the efficiency decreased to 0.8621 (Figure 4).

**Figure 5:**
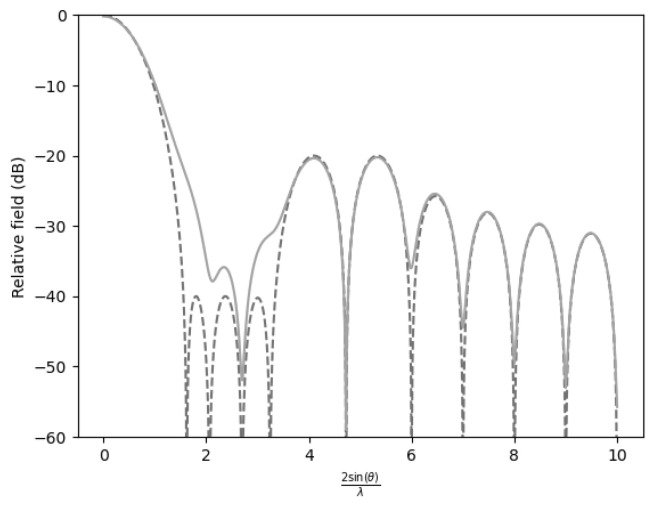
Comparison of the far-field expression (γ=1000) with the near-field expression (γ=0.5) for −20 dB SLL with $\bar{n}=6$ and the first three sidelobes at −40 dB.

Moreover, when three sidelobes were suppressed to −40 dB (Figure 5), the efficiency further decreased to 0.8320. While this efficiency remained higher than in the scenario where all sidelobes were attenuated to −40dB, a large penalty was already observed relative to the case with a single suppressed sidelobe.

**Figure 6:**
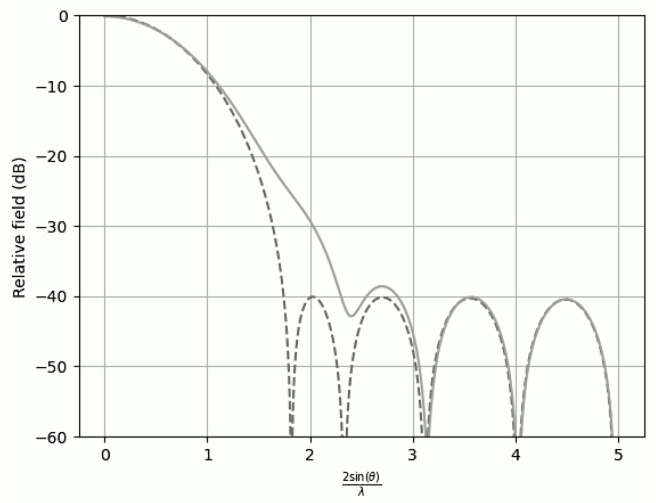
Normalized measurement distance required to recover far-field (γ=1000) using −40 dB SLL with $\bar{n}$= 11 with γ=0.5.

This behaviour confirms that the loss of efficiency becomes more significant as attenuation of sidelobes decreases to −40dB. The most significant penalty occurred when all sidelobes were suppressed to −40dB (Figure 6), with a strong effect on the overall energy distribution. Conversely, minimizing interference with the sidelobes helped maintain higher efficiency and with the best efficiency-to-lobe control trade-off was observed when only one sidelobe was adjusted to −40dB.

### Analysis of Depressed Patterns with One, Two, and Three Sidelobes Affected

As previously mentioned, in order to obtain patterns with one, two or three depressed sidelobes, Elliott’s method [22] was used to determine the roots, which were then implemented in Equation (8). The values obtained are provided in Table 2. It is important to note that reducing sidelobes leads to a decrease in efficiency. This technique enables the suppression of only the targeted sidelobes while maintaining the others at their original levels. This process ensures that the loss of efficiency is minimized.

A novel synthesis approach was formulated, and distinct performance traits were observed. Incorporation of a trade-off between main lobe intensity and sidelobe suppression requires identification of appropriate pattern specifications to optimize system operation.

Analysis of the distance required to recover the far-field pattern revealed a significant impact when one or two sidelobes were suppressed in a radiation pattern. Suppression of a single sidelobe resulted in a notable variation in the recovery distance, while elimination of the first two sidelobes further amplified this effect.

When the first sidelobe was attenuated, the normalized distance required to recover the far-field increased relative to a pattern devoid of sidelobe suppression (Table 3). However, when both the first and second sidelobes were reduced, the recovery distance underwent an even more pronounced increase. Furthermore, when the first three sidelobes were attenuated, the recovery distance increased even further.

For an error tolerance criterion of 1.0 dB, the distance required for the suppression of the first sidelobe increased to 9.7, while the suppression of the first two sidelobes increased this value to 15. The suppression of the first three sidelobes resulted in 12.5, compared to 1.5 in the absence of suppression (Table 3). It is evident that the discrepancy between these values increased as the error tolerance criterion became more stringent. For an error margin of 0.5 dB, the required distance for a pattern with the first sidelobe suppressed was 14. With the first two sidelobes suppressed, this distance was reduced to 7.5, and with the first three sidelobes suppressed, it was 5.7. In comparison, for the unmodified case, the required distance was 1.8. Finally, for an error tolerance of 0.1 dB, the required distance increased significantly, reaching 33 and 4.9 for one and two suppressed sidelobes, respectively, and 12.5 for three suppressed sidelobes, compared to 3.4 for the unaltered configuration.

These findings demonstrate that sidelobe suppression substantially alters the distance required to recover the far-field pattern. While reduction in these sidelobes can enhance specific aspects of the radiation pattern, it concomitantly imposes constraints on the required distance, a factor that must be taken into account in the design and optimization of antenna systems.

## 5. Conclusions

We have demonstrated that the reduction or suppression of one, two, or three interior lobes in a Taylor-type pattern results in enhanced efficiency, relative to reducing almost all lobes, which yields the lowest efficiency. However, the distance required to recover the pattern is significantly greater than when all lobes are depressed. Furthermore, the required distance is greater when only one lobe is suppressed than when two are suppressed. Several promising research directions for near-field communications, such as the improvement in the Rayleigh distance and the development of hybrid-field transmission techniques, are also highlighted. These areas are expected to drive further innovations in 6G near-field MIMO systems.

## Figures and Tables

**Table 1:** Normalized measurement distance required to recover the far-field for error acceptance criteria of 0.1 dB, 0.5 dB, and 1.0 dB.

SLL (dB)	Error 0.1 dB	Error 0.5 dB	Error 1.0 dB
−40	9.0	4.2	3.0

**Table 2:** Values of *Zn* for one, two and three depressed inner sidelobes at −40 dB, for −20 dB SLL, and $\bar{n}$ = 6, according to Elliott’s iterative method.

Root Number	*Zn* for First Side Lobe Depressed	*Zn* for First Two Side Lobes Depressed	*Zn* for First Three Side Lobes Depressed
1	1.6408	1.9276	1.6235
2	1.3860	1.5346	2.0662
3	2.7762	2.3839	2.6999
4	3.8145	3.7234	3.2456
5	4.8740	4.8313	4.7244

**Table 3:** Patterns with different sidelobe depressions.

Error Criterion Acceptance (dB)	γ for First Sidelobe Depressed	γ for First Two Sidelobes Depressed	γ for First Three Sidelobes Depressed	γ for No Sidelobe Depressed
1.0	9.7	4.9	4.1	1.5
0.5	14	7.5	5.7	1.8
0.1	33	15	12.5	3.4

## Data Availability

The datasets used and/or analyzed during this study are available from the corresponding author upon reasonable request.

## References

[1] Selvan K.T., Janaswamy R. (2017). Fraunhofer and Fresnel Distances: Unified Derivation for Aperture Antennas. IEEE Antennas Propag. Mag..

[2] Capozzoli A., Curcio C., D’Agostino F., Liseno A. (2024). A Review of the Antenna Field Regions. Electronics.

[3] Abdallah M., Sarkar T., Palma M.S., Monebhurrun V. (2016). Where Does the Far Field of an Antenna Start? [Stand on Standards]. IEEE Antennas Propag. Mag..

[4] Skulkin S.P., Turchin V.I., Kascheev N.I. (2018). Range Distance Requirements for Large Antenna Measurements for Square Aperture with Uniform Field Distribution. IEEE Antennas Wireless Propag. Lett..

[5] Yaghjian A.D. (2025). Generalized Far-Field Distance of Antennas and the Concept of Classical Photons. IEEE Trans. Antennas Propag..

[6] Taylor T.T. (1955). Design of Line-Source Antennas for Narrow Beamwidth and Low Side Lobes. Trans. IRE Prof. Group Antennas Propag..

[7] Elliott R.S. (2003). Antenna Theory and Design.

[8] Dolph C.L. (1946). A current distribution for Broadside Arrays Which Optimizes the Relationship Between Beamwidth and Side Lobe Level. Proc. IRE.

[9] Hansen R.C. (1984). Measurement Distance Effects on Low Sidelobe Patterns. IEEE Trans. Antennas Propag..

[10] Hansen R.C. (1992). Array Pattern Control and Synthesis. Proc. IEEE.

[11] Hansen R.C. (1992). Measurement Distance Effects on Bayliss Difference Patterns. IEEE Trans. Antennas Propag..

[12] Brégains J.C., Ares F., Moreno E. (2003). Effects of Measurement Distance on Measurements of Symmetrically Shaped Patterns Generated by Line Sources. IEEE Antennas Propag. Mag..

[13] Torrado-Puime A., López-Martín M.E., Rodríguez-González J.A., Ares-Pena F.J. (2024). Measurement Distance Effects on *ϕ*-Symmetric Taylor Patterns with Optimal Transition Integer *n* and with Reduced Inner Sidelobes. Sci. Rep..

[14] Torrado-Puime A., López-Martín M.E., Rodríguez-González J.A., Ares-Pena F.J. (2025). Null-filling effects over measurement distances on circular Taylor patterns with optimal transition integer and with reduced inner sidelobes. Sci. Rep..

[15] Guo Y., Guo C.A., Li M., Latva-aho M. (2025). Antenna Technologies for 6G – Advances and Challenges. IEEE Trans. Antennas Propag..

[16] Zhang J., Xiao M., Huang Y., Choi J., Heath R.W., Letaief K.B. (2022). Near-Field MIMO Communications for 6G: Fundamentals, Challenges, Potentials, and Future Directions. IEEE Commun. Mag..

[17] Zhang H., Shlezinger N., Guidi F., Dardari D., Eldar Y.C. (2023). 6G Wireless Communications: From Far-Field Beam Steering to Near-Field Beam Focusing. IEEE Commun. Mag..

[18] Liu M., Zhang Y., Jin Y., Zhi K., Pan C. (2024). Towards Near-Field Communications for 6G: Challenges and Opportunities. ZTE Commun..

[19] Liu Y., Ouyang C., Wang Z., Xu J., Mu X., Swindlehurst A.L. (2025). Near-Field Communications: A Comprehensive Survey. IEEE Commun. Surv. Tutor..

[20] Hansen R.C.E. (1985). Microwave Scanning Antennas.

[21] Walter C.H. (1965). Traveling Wave Antennas.

[22] Elliott R.S. (1976). Design of Line Source Antennas for Sum Patterns with Sidelobes of Arbitrary Heights. IEEE Trans. Antennas Propag..

[23] (2014). IEEE Standard for Definitions of Terms for Antennas.

[24] López-Álvarez C., López-Martín M.E., Rodríguez-González J.A., Ares-Pena F.J. (2023). Maximizing Antenna Array Aperture Efficiency for Footprint Patterns. Sensors.

[25] Balanis C.A. (1997). Antenna Theory: Analysis and Design.

[26] Stutzman W.L., Thiele G.A. (1998). Antenna Theory and Design.

